# We’re going bananas! The effectiveness and safety of banana puree as an intervention for improving oral feeding in a level IV NICU

**DOI:** 10.1038/s41372-026-02765-z

**Published:** 2026-07-07

**Authors:** Cara Beth Carr, Sheri E. Ricciardi, Saya Bery, Rebecca Levin, Deanne Wilson-Costello, Gulgun Yalcinkaya, Rita M. Ryan

**Affiliations:** 1 University of North Carolina at Chapel Hill, Chapel Hill, NC USA; 2 Rainbow Babies and Children’s Hospital, University Hospitals of Cleveland Medical Center, Cleveland, OH USA; 3 Case Western Reserve University, Cleveland, OH USA

**Keywords:** Nutrition disorders, Nutrition therapy

## Abstract

**Objective:**

To evaluate the safety and feasibility of banana puree thickener in neonatal intensive care unit (NICU) patients with dysphagia.

**Study design:**

This is a single-center, retrospective study of 43 infants (median gestational age 30 weeks (w), 1320 g birthweight) who received banana puree for dysphagia between 2015 and 2023. Results were analyzed using summary statistics and Wilcoxon signed-rank tests.

**Results:**

Banana intervention (BI) was introduced at median postmenstrual age 42w after inability to attain full oral feedings. Oral intake percentage improved from a median of 50% (IQR 17, 100) to 100% (45, 100, *p* = 0.0002) seven days after BI started. Bananas successfully thickened breast milk; 23 (53%) infants were discharged with BI; of those not using BI at discharge, 15 gained short-term benefit and no longer required BI. No patients stopped BI due to adverse effects.

**Conclusion:**

This study demonstrated that banana puree for dysphagia is safe, feasible, and improved oral feeding.

## Introduction

Premature and/or medically complex infants have significant feeding difficulties arising from an interplay of extreme prematurity, low birth weight, chronic illness, prolonged intubation, comorbidities, and other conditions that result in the delayed introduction of oral feeding [1–12]. Studies have shown that over 65% of infants born between 29 and 33 weeks (w) gestational age (GA) have prolonged hospitalizations, primarily related to insufficient oral feeding [13]. Neonatal occupational therapists (OTs) and speech-language pathologists (SLPs) recommend thickened liquids only after optimizing standard interventions such as ensuring neurobehavioral maturation and feeding readiness, utilizing ultra slow-flow nipples and bottle systems, optimizing positioning, and maximizing therapy frequency [7, 14–16].

While thickened feeds is an accepted industry standard in pediatrics, thickeners remain limited in neonates due to concerns of necrotizing enterocolitis (NEC) and other complications that resulted in the removal of commercial thickeners from the market [17, 18]. Therefore, a clinical need emerged at our institution to find an acceptable alternative for thickening in infants >36w postmenstrual age (PMA), after exhausting all other standard-of-care options. The search for a viable thickener that was sweet, homogenous, safe, and effective led our team to explore the use of banana puree as an innovative alternative. The unique type of starch found in bananas has a dense hexagon pattern with double helices, which may defend amylase from quickly breaking down the starch while allowing a slight thickening effect when using human milk and remaining easily digestible [19]. This characteristic and the known nutrient composition of bananas led to the implementation of banana puree for dysphagia at our institution [20].

## Methods

### Study design and participants

This was a single-center retrospective study conducted at University Hospitals (UH) Rainbow Babies and Children’s Hospital/Case Western Reserve University. It included patients who were ≥36w PMA and received banana puree as a feeding intervention in our NICU between 2015 and 2023. These patients were identified by neonatal feeding therapists (occupational therapists) as candidates for banana puree intervention (BI) after a failure to improve oral intake with other standard feeding strategies. Infants were not considered BI candidates if they had a known latex allergy, banana allergy in caregiver/sibling, confirmed hyperkalemia, or history of surgical NEC [21]. The lead neonatal OT (author SR) maintained a prospective log of BI patients, which was cross-referenced with a complete census of all NICU patients between the years of 2015 to 2023 to ensure correct patient identification. This retrospective study was approved by the UH Institutional Review Board.

### Data collection

Data were collected via retrospective chart review of the electronic medical record and maintained using UH Research Electronic Data Capture (REDCap) [22, 23]. Patient demographic information included gestational age (GA), birth weight, and sex. To characterize infants’ feeding patterns and the effect of the BI, we collected PMA at first oral feeding, PMA at first banana intervention (BI), reason for initiation of BI, oral intake (ml/kg/day) before and after BI, composition of enteral feeds (human milk vs formula), number of OT feeding sessions that mention banana intervention, growth parameters, and discharge characteristics. As is routine in our unit, the occupational therapists trended each patient’s 24-h saturation histograms and charted significant events; per the implementation protocol, they would have stopped the intervention if any concerns arose. The occupational therapy and primary team documentation were reviewed to determine the reason for discontinuation of intervention and to evaluate for any perceived adverse effects. For this study, full oral intake volume was defined as a minimum of 120 ml/kg/day of human milk or formula. To assess the potential adverse effects of BI, we collected data on growth parameters, respiratory support, and the need for NPO status before and after BI. Each baby was carefully assessed by an OT before and after BI sessions, with attention to both saturation profile measurements to monitor for possible profile shifts suggesting hypoxemia and for the number of apnea/bradycardia/desaturation events requiring a change in respiratory support. Specific time points assessed for each patient included seven days prior to banana intervention (Day −7), the day prior to the start of BI (Day −1), the day of the first banana use (Day 0), seven days after implementing bananas (Day 7), and fourteen days after implementing bananas (Day 14).

### Data analysis

Categorical data were analyzed using descriptive summary statistics including frequency and percentages. Continuous data were analyzed using descriptive statistics including median and interquartile range, given non-normally distributed data. Wilcoxon signed-rank test was used to compare paired non-normally distributed measures before and after BI. This study examined all available subjects (over an eight-year period) to report on the feasibility and safety of BI, and thus, a sample size calculation was not performed for this study.

### Recipe for banana puree intervention

Prior to the publication of the International Dysphagia Diet Standardization Initiative (IDDSI) classification [24], author SR developed a basic recipe to achieve a change in thickness between “thin” and “slightly-thick” using a percentage of banana to the total volume of feed. Different amounts of a commercially available banana puree (Gerber Natural for Baby Banana 1st Foods-Stage 2 (Arlington, VA, USA)) were trialed to achieve the desired consistency. At our institution, the IDDSI classification was used to compare our percentage of banana used to IDDSI levels 1 and 2 (“slightly thick” and “mildly thick”); however, we frequently found our thickened substrate reached a level between “thin” and “slightly thick”. To assess for these smaller changes in thickness, we compared time (seconds) needed for the substrate without banana to empty a syringe to other substrates with various amounts of banana. For example, if a substrate without any banana emptied the syringe at seven seconds, and a 15% banana thickened substrate emptied at nine seconds, a slight change in thickness was observed even if it did not reach the level of IDDSI “slightly-thick”. Banana to substrate percentages began as low as 10% and increased as high as 20% as deemed clinically necessary by author SR.

The BI Recipe:

- Step 1: Calculate the banana puree amount based on desired percentage to the total feed (e.g., 15% banana/milk ratio, such as 4.5 mL banana to 30 mL of milk).
- Step 2: Warm human milk/formula.
- Step 3: Measure the desired amount (e.g., 4.5 mL) of banana puree with a syringe.
- Step 3: Add the banana puree (chilled or room temperature) to the warmed milk/formula.
- Step 4: Shake bottle gently to combine. The bottle does not need to be rewarmed after the banana puree is added.
- Step 5: Feed bottle to infant.

## Results

### Patient characteristics

The median GA of infants who received banana puree was 29.7 weeks with a median birth weight of 1320 g (Table 1). PMA at first oral feeding attempt occurred at ~38 weeks’ gestation. Approximately four weeks later, patients were first introduced to banana puree (median PMA 41.7 weeks). Patients received a median of 9 days of BI, with a median of 6 therapy encounters that incorporated feeding with the banana intervention (BI). The remaining feedings were done by bedside nurses or caregivers.

**Table 1:** Patient characteristics and safety data.

Patient characteristics	Infants (*n* = 43)
Gestational age (weeks)	29.7 (25.4, 37.6)
Birth weight (grams)	1320 (810, 2890)
Female sex	22 (51%)
PMA at first oral feeding (weeks)	37.9 (34.9, 42.9)
PMA at first BI (weeks)	41.7 (39.6, 46.3)
Number of days infants received BI	9 (6, 16)
Number of infants discharged with BI	23 (53%)
Safety of BI	
Infants placed on increased respiratory support during a feed	1 (2%)
Infants requiring NPO status due to BI	0 (0%)
Effect of banana intervention on oral feeding volume	
Enteral feed volume (PO + NG) before BI (mL/kg/day)	145 (126, 155)*
Enteral feed volume (PO + NG) Day 7 of BI (mL/kg/day)	134 (113, 150)*
PO feed volume before BI (mL/kg/day)	68 (17, 128)*
PO feed volume on Day 7 of BI (mL/kg/day)	117 (51, 143)*
Percentage of total enteral feeds taken PO before BI	50 (17, 100)*
Percentage of total enteral feeds taken PO on Day 7 of BI	100 (45, 100)*

Data represented as median (IQR) or frequency (percentage).

*BI* banana intervention, *NPO* nil per os, nothing by mouth, *PMA* postmenstrual age, *OT* occupational therapy (neonatal feeding therapist), *PO* by mouth, *NG* nasogastric.

*P ≤ 0.05 by Wilcoxon signed-rank test.

### Indications for banana intervention

Neonatal OTs identified candidates for BI based on observed feeding behaviors thought to be contributing to poor oral intake, such as oral-pharyngeal incoordination, reflux, and oral aversion. The majority (53%) of patients started BI due to general incoordination issues with oral feeds that persisted outside of standard-of-care interventions and were not overtly due to a lack of infant cues or significant delays in maturation. Of note, BI was not used as an attempt to advance the typical neuromaturation time for feeding skill progression and was only utilized in the presence of clinical signs of dysphagia.

### Feeding type and amount

**Fig. 1:**
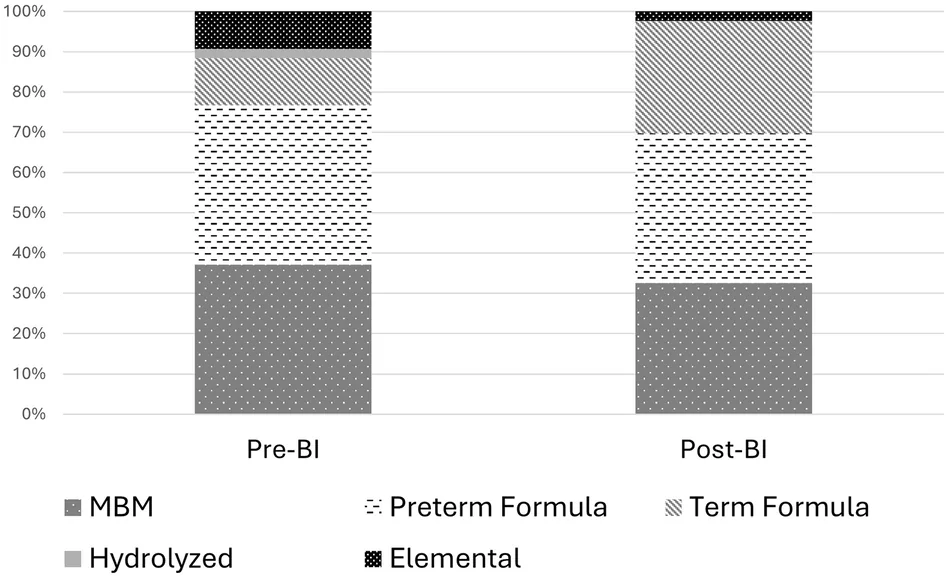
Primary component of infants’ feeds. Approximately the same percentage of infants used maternal breast milk as the primary component of their feeds before (*n* = 16, 37%) compared to after (*n* = 14, 33%) receiving the banana puree intervention. No infants received DBM as their primary component of feeds. (MBM maternal breast milk, DBM donor breast milk).

Prior to starting BI, 20 patients (47%) were receiving some amount of maternal human milk, with 16 of those patients (37%) receiving human milk as the majority of their feed composition (Fig. 1). More commonly, feeds consisted primarily of preterm, term, or elemental formula. The percentage of patients receiving human milk as the primary component of feeds remained stable after the BI (Fig. 1). At discharge, the percentage of patients receiving any amount of human milk decreased minimally to 42% (*n* = 18).

**Fig. 2:**
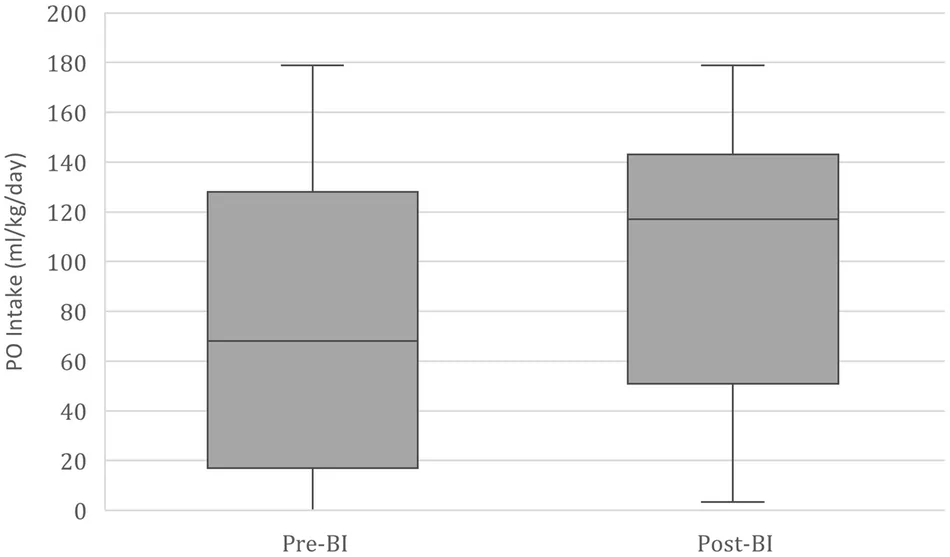
Change in oral intake after BI. The amount of total enteral feeds taken PO significantly increased after the banana intervention (BI) from a median of 68 ml/kg/day before the BI to 117 ml/kg/day after BI (*p* = 0.003, Wilcoxon signed-rank test).

At the start of BI, patients were receiving a median of 145 mL/kg/day of enteral feeds and 68 mL/kg/day of oral (PO) feeds (Table 1). By Day 7 of BI, the median amount of oral feeds increased to 117 mL/kg/day (Fig. 2; *p* = 0.003, Wilcoxon signed rank test), with 79% (*n* = 34) of patients achieving 120 mL/kg/day of oral feeds. By Day 7 of BI, the median percentage of PO feeds increased from 50% to 100% (*p* = 0.002, Wilcoxon signed rank test). Fifteen patients were taking full PO feeds before initiating BI. These patients started BI due to general coordination issues (*n* = 5), apnea/bradycardia/desaturation events during PO feeds (*n* = 4), reflux (*n* = 5), and an unknown reason (*n* = 1).

### Safety parameters - weight gain

**Fig. 3:**
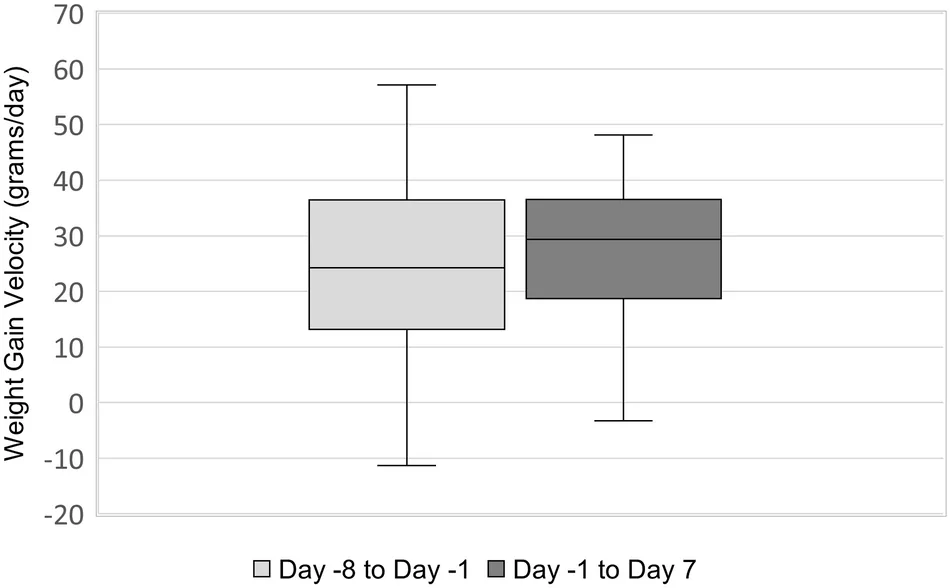
Weight gain velocity before and after banana intervention. There was no decrease in weight gain velocity during the week before BI (24 grams/day) and the week after BI (29 grams/day) .

Weight gain velocity was tracked due to the concern that banana puree could be supplanting high caloric feeding, resulting in a slower weight gain velocity. Infants showed no statistically significant difference in weight gain velocity in the week prior to and after initiation of bananas (Fig. 3).

### Respiratory status changes

**Fig. 4:**
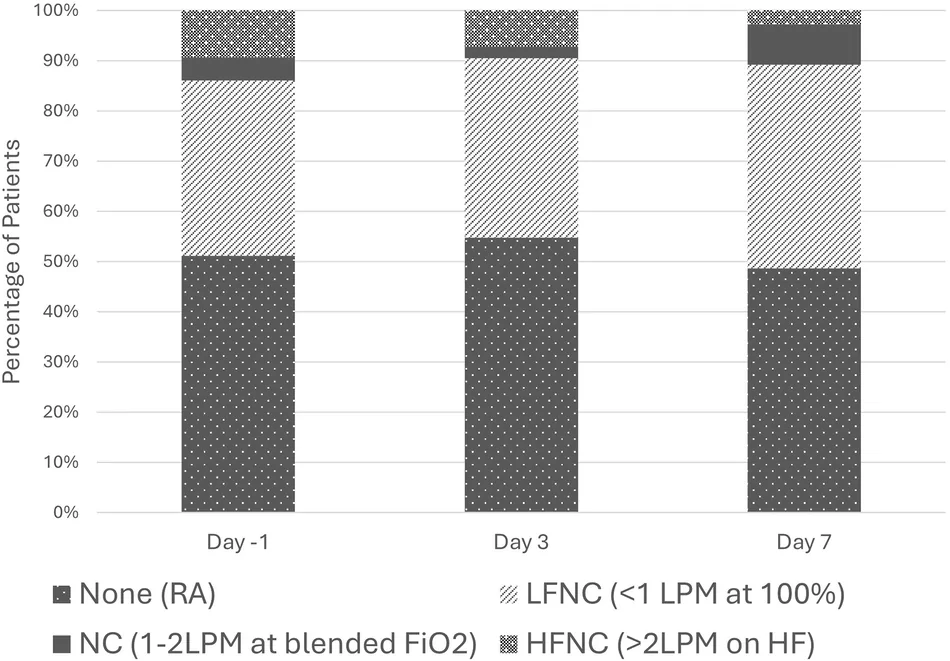
Respiratory support before and after BI. To assess potential safety concerns of the banana puree intervention, infants’ respiratory support level was assessed for any change before (Day −1) BI, shortly after starting BI (Day 3), or one week after starting BI (Day 7). Infants showed no significant change in respiratory status during this time.

Patients were on varying types of respiratory support before starting BI, including room air, nasal cannula (NC) (1-2 LPM), and high flow nasal cannula (HFNC) (>2LPM) (Fig. 4). There were no perceived adverse respiratory effects of the BI leading to early discontinuation of the intervention. One patient trialed increased respiratory support in an effort to improve feeding endurance, but this was unrelated to the BI process. In 2021, our institution developed a new protocol that allowed infants to start PO feeds while on HFNC [25]. Figure 3 demonstrates that most patients requiring HFNC on Day −1 of BI were able to be weaned to lower respiratory support by Day 7 of BI, indicating that BI does not delay weaning of respiratory support in this subgroup of patients. No patient required NPO status due to BI.

### Discontinuing banana intervention and discharge

Approximately half (*n* = 23, 53%) of patients were discharged home with BI. Most patients not using BI at the time of discharge discontinued the intervention during their hospital admission after successfully achieving improved and consistent oral intake with BI (*n* = 15). Additional reasons for discontinuing BI included parental preference (*n* = 1), patient refusing all oral feeds (*n* = 1), and lack of functional gains (*n* = 1), resulting in a referral for a swallow study and gastrostomy tube placement. One patient discontinued BI due to no perceived benefit of the intervention; no patients discontinued BI due to an adverse response/reaction. At discharge, 81% (*n* = 35) of patients who received BI were taking 100% oral feeds. Three patients (7%) were discharged with a gastrostomy tube and five patients (12%) with a nasogastric tube. Of the eight patients discharged with feeding tubes, three patients had successfully reached 120 mL/kg/day of oral feeds with BI but were unable to sustain their intake. Of the fifteen patients who started BI for reasons other than failing to achieve full PO feeds, 63% (*n* = 10) were discharged home with BI.

## Discussion

This retrospective study demonstrates the feasibility and safety of banana puree for dysphagia in NICU patients. Infants with difficulty swallowing thin liquids showed significant improvement within seven days of banana puree added to formula or human milk under the supervision of a neonatal feeding therapist.

While studies have shown improved oral transit and swallowing with increased viscosity of feeds, there is no evidence for a specific viscosity/thickness that guarantees clinical effectiveness [26]. Our study showed that clinical improvements were noted using a 10–20% banana puree to total volume recipe. Current research has examined the amount of banana puree in determining IDDSI levels of slightly thick (Level 1) or mildly thick (Level 2) when attempting to achieve those desired levels of thickness [27]. Likewise, the 2021 study by Brooks et al. performed rheology testing on liquids thickened with banana puree to measure viscosity at various shear rates and temperatures. The results revealed that banana puree was comparable to gum and starch based commercial thickeners in its shear thinning behavior and effectively thickened expressed human milk at various temperatures and maintained that thickness over time [28, 29]. These benefits, along with ease of accessibility, nutritional value, taste, and ability to blend homogeneously with human milk and formula are key factors in pursuing banana puree as a novel thickening agent. In our study, the addition of banana puree as a thickener at an earlier PMA than currently recommended with carob bean gum thickeners (>42 wk) allowed us to sustain human milk provision at discharge (42%) in this complex patient population that may have otherwise been compromised [30]. The thickness achieved by our banana recipe often qualifies as between “thin” and “slightly thick” IDDSI classifications, and its small change in thickness was only discernible by timed syringe tests. Based on the results of our study, small changes in thickness can result in clinical effectiveness; it may be that more sensitive IDDSI methods could be explored in future studies.

It is notable when looking at the interquartile range (IQR) that there is a wide distribution of gestational age in our BI population, with 25% of patients born before 25w GA and 25% of patients born at term. This demonstrates that BI was successful across a wide variety of etiologies for dysphagia despite this non-homogeneous cohort.

In our cohort, there were about four weeks between the first oral feeding and introducing banana purees. During this time, our feeding team tried other standard dysphagia interventions including maximizing the use of slow flow nipples/bottles, pacing, inclined side-lying, and consistent therapeutic support to staff and caregivers. When initiating BI, the infants’ PO intake had plateaued at ~50%; however, after just one week of BI, the median PO intake increased to 100%. Since each infant served as their own comparison, we intentionally focused on data points one week apart to determine the short-term effects of BI while minimizing the confounding aspect of neurodevelopmental maturation over time. Although there was a significant decrease in the total amount of enteral intake from 145 ml/kg/day before BI to 134 ml/kg/d after BI, this is likely reflective of more infants transitioning to PO ad lib given the significantly increased amount of PO intake. Additionally, the majority of patients who initiated BI for reasons other than failure to reach full PO feeds were discharged home with BI, which indicates a perceived benefit by the clinical team for indications such as general oral coordination skills, apnea/bradycardia/desaturation events during PO feeds, and reflux. Not only do the data indicate a significant short-term impact of the intervention, but 80% of patients had sustained oral feeding success at the time of discharge, signifying a continued long-term effect as well. The results suggest there may be an opportunity to consider BI earlier in babies who are showing strong readiness cues yet are having trouble gaining oral feeding skills. In addition, the median postmenstrual age at first oral feeding was 38 weeks. This suggests that the babies in this cohort had significant comorbidities or respiratory support delaying their first oral feeding attempt. Specific comorbidities were not detailed for this study, but future studies may benefit from assessing these comorbidities to identify subpopulations of patients who may receive the greatest benefit from this intervention.

A sample size needed to detect a difference between 50 and 100 percent PO within seven days with a standard deviation of 35 would be six pairs of infants. In this study, each baby served as their own control pre/post BI and was their own comparator; our study includes 37 babies in the comparison of percentage PO intake before and seven days after BI; six babies were discharged home before the seven day point after BI and were not included in this particular analysis.

Our safety data regarding weight gain velocity, respiratory support, and perceived adverse effects support the safety of BI as a dysphagia intervention. Adding banana puree to formula or human milk has the potential concern of displacement of calories, which may have a negative nutritional impact if using BI with every feed. We did not see a change in weight gain velocity with the implementation of BI during its first week. However, we recommend continued close monitoring of growth parameters if infants are to continue BI for an extended period. There was also no concern for increased respiratory support requirements due to BI. Infants were clinically monitored for any perceived adverse reactions secondary to BI at the time of the intervention during daily medical rounds and routine lab work, and no perceived adverse reactions were noted. Additionally, the percentage of banana to substrate intake that these patients received (~15%) is in line with the recommendations from Brinker et al. to restrict banana supplementation to 15% banana to daily total fluid volume to avoid excessive potassium intake [27].

The novel aspect of our banana puree intervention emphasizes the importance of a multidisciplinary approach during and after inpatient hospitalization. Since many patients are discharged using banana purees, close coordination with the patient’s outpatient team is necessary. Detailed discharge instructions are provided to the pediatrician, and the infants are followed by their pediatrician, the outpatient neonatology clinic, and the outpatient feeding team. At our institution, the feeding team, with close coordination with the primary neonatologist or pediatrician, primarily determines the timing for discontinuing banana purees.

This retrospective study was limited by accessing patients found in our institution’s previously used Electronic Medical Record (EMR). Therapists and physicians who were present across the time-period of this study provided anecdotal context; however, data collection was limited due to the search features of the prior EMR. In addition, the lack of a control group limits the ability to analyze long-term outcomes due to potentially confounding factors such as typical neuromaturation and oral feeding skill progression. As we do not routinely assess infants with videofluoroscopic swallow studies (VFSS) before beginning therapist-guided thickening trials in our NICU, this may be a potential limitation for generalizability to units that do routinely utilize VFSS.

Future studies are needed to examine the long-term efficacy and superiority of using banana purees compared to other thickening methods in infants less than six months of age. This study has found banana puree to be a safe and feasible option for feed thickener; however, we recommend additional data be obtained from prospective, controlled studies with a matched control group to allow a more in-depth assessment of its benefits and risks. Additionally, these future studies should include longitudinal follow-up data to better assess feeding post-discharge and potential late adverse effects. Criteria for using banana puree as a thickener should be outlined in clinical practice guidelines (Supplemental Text) that can be replicated between institutions, allowing for multi-institutional trials and quality improvement initiatives. Diagnostic studies, including fiberoptic endoscopic evaluation of swallowing (FEES), manometry, video fluoroscopy, and pre-post intervention pneumograms, could further assess the safety and efficacy of banana puree as an intervention for dysphagia and reflux. Since our study showed a potential benefit for dysphagia using just a slight change in thickness of banana puree, it would be prudent to evaluate more sensitive methods of testing viscosity per IDDSI guidelines to capture the small changes that may benefit the neonatal population. Finally, prospective studies with a true matched control group can assess whether BI influences the need for g-tube placement or time to discharge. However, BI appears to be safe and could be used in our described method while awaiting additional studies.

## Conclusions

Commercial banana puree is a safe and feasible thickener option for improving dysphagia and oral feeding skills in neonates. Banana puree thickener may help infants achieve full oral feedings prior to discharge without adverse effects and should be considered in infants whose feeding skills are not progressing with standard feeding interventions. This pilot data is provided to serve as the basis for a future randomized controlled trial.

## Supplementary information

Supplemental Text

## Data Availability

The datasets generated during and/or analysed during the current study are available from the corresponding author on reasonable request.
