# Supplementary material for: We’re going bananas! The effectiveness and safety of banana puree as an intervention for improving oral feeding in a level IV NICU
Source: J Perinatol. 2026 Jul 7;46(9):1635–40. doi: 10.1038/s41372-026-02765-z (PMC13588743; doi:10.1038/s41372-026-02765-z)
Supplement: Supplementary file 1 [file 41372_2026_2765_MOESM1_ESM.docx]

**Supplemental Text**

**Rainbow Babies and Children’s Hospital**

**Neonatal Therapy Clinical Practice Guideline for Use of Banana Puree as a Thickening Agent in the NICU in Infants >37 Weeks PMA**

**Rationale for thickening:**

1. Convalescing preterm and medically complex infants often have difficulty swallowing thin liquids resulting in poor oral intake.^[1]^
2. Studies have shown improved safety and coordination with slightly thickened oral feedings.^[2]^
3. Banana thickened liquids may enhance sensory/olfactory/gustatory pathways effective in promoting improved bolus cohesion and slowed oral pharyngeal flow needed for safe, efficient feeding.
4. Thickening can be a temporary intervention or can become part of the longer-term feeding plan if nutritionally appropriate and clinically effective, safe under the supervision of a feeding specialist.^[3],^^[4],^^[5],^^[6]^

**Rationale for banana puree:**

1. Homogenously blends with breast milk and formula.^4,5^
2. Effectively thickens breastmilk with stable viscosity across a typical feeding.^4,5^
3. The only effective form of thickening breastmilk in infants less than 42 week postmentrual age (PMA) infants. ^4-6^
4. Ease of accessibility for home use as needed.
5. Sweet taste-similar to breast milk.

**Indications for use:**

1. Infants corrected to 37+ weeks PMA with oral feeding difficulty despite trial of all standard of care interventions for greater than one week and per Neonatal Feeding Therapist recommendation.
2. Clinical or diagnostic recommendation for thickened feeding trial.
3. Potential for mild reflux mitigation especially to preserve exclusive breast milk diet.
4. Unsuccessful or contraindicated thickening agents (oatmeal/rice/Gelmix).
5. Prudent use for oral stim/aversion issues and entraining swallowing in post term infants.

**Exclusion/Contraindications:**

1. Infants below 37 weeks PMA.
2. Patients with risks for Latex Allergy due to possible Oral Allergy Syndrome (cross reactivity concerns) (e.g., Myelomeningocele).
3. History of surgical NEC – must be cleared by surgical team/medical team
4. Medical NEC - must be cleared by medical team
5. Parent or sibling allergy to banana.
6. Concern for significant hyperkalemia/hyperglycemia baseline disorders - confirm with medical team.

**Clinical Behaviors for Initiating Banana:**

After discussion with the multidisciplinary team, inclusion criteria are met, and all standard of care feeding strategies have been maximized, consider starting banana intervention on infants with the following clinical sequelae:

1. Incoordination of suck swallow breath sequencing
2. Minor desaturations with oral feeding
3. Dynamic stridor, stertor that is only present with feeding
4. Cough, breath holding, watery eyes, or suspected s/s of transient aspiration
5. Reflux mitigation on exclusive breastmilk diet
6. Oral aversion

**Clinical Behaviors for Discontinuing Banana:**

1. No improvement in coordination with feeding after ~ 3 days
2. Emesis or worsening feeding skills
3. New onset hyperkalemia
4. If the intervention is successful: once 100% oral feeding has been achieved for a minimum of one week, consider reducing percentage of banana with reassessment from feeding therapist

**Implementing Banana Thickened Trials:**

1. Initial assessment with a small exposure of banana puree added to base liquid (formula or human milk) **should only be assessed and implemented under the guidance of a Neonatal Feeding Therapist.**
2. Therapy assessment should confirm efficacy, level of thickness, appropriate nipple/bottle, and infant specific recommendations
3. The rationale for use, impact on overall nutritional goals, and patient tolerance to intervention should be discussed with the dietitian, feeding therapist, and primary medical team.
4. The Neonatal Feeding therapist will determine the banana recipe using a % of banana to base substrate.
5. Once indicated, consider progression as follows using infant driven feeding readiness and quality scores: Recommend starting with 15% banana and going either up or down depending on patient needs (10-20% range).
   a. Day 1: 15% banana to substrate ratio x 1-2 feeds with therapy
   b. Day 2: 15% banana to substrate ratio x 3 feeds
   c. Day 3: Proceed with clinical trial up to 50% full volume
   d. Day 4: Advance to full feeds with < 20% banana to total volume
   e. If using 20% banana to achieve optimal thickness-discuss with dietitian and medical team. Consider nutritional displacement impact with 20%. Will depend on base substrate and the individual patient’s nutritional needs. Monitor labs, growth and individual patient needs closely.
6. Monitor respiratory changes, A/B/D events, saturation profiles, and MD/APP clinical exam daily during banana trial. If there is no clinical improvement with a trial of 20% banana to substrate ratio, refer infant for formal imaging swallow study.^[7]^

**Mixing Instructions:**

a. Warm human milk or formula separately
b. Use Stage 2 Gerber banana puree (brand provided and tested at Rainbow)
c. Measure desired amount of banana with syringe
d. Add banana puree to warmed milk
e. Do not rewarm once banana is added
f. Gently shake to mix
g. Offer with bottle and desired nipple (depending on % of banana added and degree of infant incoordination -may not need to go up on nipple flow)
h. Thickened substrate will stay stable across feeding time of ~20-30 min ^3^
i. If using human milk: Gavage any amount not taken orally to prevent waste of human milk. Up to 20% of the banana added can go through a 5 French feeding tube without clogging.
j. If using formula: discard the thickened substrate and gavage non thickened formula.
k. After opening, store bananas in an airtight container. You can use Volufeed or pumping container with cap. Add patient label, document ‘banana’, and expiration date of 48 hours. (e.g., banana, exp. 6/23/25 @ 1200)
l. Optional: Draw up desired amount of banana in individual syringes at time of opening and label/date needed syringes for 24-48 hours.
m. Discard any unused banana after 48 hours.

**Desired Outcomes:**

1. Improved qualitative feeding behaviors: less distress or incoordination, improved quality scores
2. Improved physiological stability (decreased A/B/D’s with feeding).
3. Increased oral feeding volume and efficiency.
4. Improved caregiver success with feeding (infant stability and efficacy).
5. Improved oral intake within 7 days of initiation.

**Clinical Monitoring:**

1. Discussion in medical rounds daily.
2. Monitor for changes in bowel habits (frequency/quality).
3. Follow weekly weight gain trends.
4. Monitor 24-hour saturation profiles, note any A/B/D’s surrounding feeding.
5. Discuss weekly labs such as K+, BUN
6. A 15% banana to substrate ratio should be within acceptable limits nutritionally and medically, however individual patient needs may vary. ^6^
7. See **Appendix 1** for Nutrient Composition of banana puree^6^ and (<https://medical.gerber.com>).

**Thickness Assessment: (Modified IDDSI testing:** [www.iddsi.org](http://www.iddsi.org))

1. If using IDDSI methodology to assess thickness and the mixture tests “thin” complete the 10-cc syringe test and **note the time** that the substrate empties the syringe.
2. For example, note if thickened substrate empties the syringe in 6 seconds without banana and 8 seconds with 15% banana, this will indicate a slight change in thickness that would not otherwise be reflected in the current IDDSI testing instructions.
3. **Slight changes in thickness will test “thin” in many cases and may still have a therapeutic effect.**

**Formal Diagnostic Testing**:

4. Refer for videofluoroscopic swallow study (VFSS) if unable to achieve clinically significant benefit in up to 20% banana use.
5. **During swallow study:** **Replicate exact diet**. **Use the desired % of banana and breastmilk/ formula with barium suspension or powder**. Banana testing with actual substrate will test differently than commercial Varibar in many cases. Use the least amount of barium powder needed.
6. If standard levels of thickness are desired, (IDDSI targets of Level 1 and 2) recipes have been established however should be verified^.6^
7. Do **not** add more than 20% banana to total volume unless volume is intentionally limited.
8. **Therapeutic volumes** of up to 1:1 banana /substrate can be used judiciously to develop skill as a recommendation from the VFSS but should be used in very small volumes and under the direction of a neonatal feeding therapist.

**Potential contraindications and rationale to discontinue:**

1. Increased abdominal girth or significant change in stooling
2. New onset rash
3. Overt clinical distress: worsening feeding incoordination, cough, gag, disinterest, refusal behaviors, or shift in 24 hours saturation profile using histogram readings
4. No appreciable improvement in feeding behaviors after 3-5 days
5. Abnormal lab values (increasing K+) (Note this can be test procedure error or from other metabolic reasons). Hold banana puree intervention until discussion with MD.
6. Nutritional concerns such as weight loss or excessive weight gain. Follow closely with dietitian.
7. If oral thrush is present or develops, discuss with team if banana would be a temporary contraindication.

**Weaning Process^[8]^**

1. **If weaning as inpatient**: Once patient has achieved full oral feeds for one week, trial wean by decreasing banana by ~5% weekly with feeding therapist recommendation.
2. **If weaning as outpatient:** General recommendation is for no changes for ~2 weeks post discharge unless under the direction of the pediatrician or home feeding team. A typical outpatient wean consists of a 10% every 2 weeks and is managed by the home feeding team.
3. Refer for diagnostic study if any clinical sequelae are noted.
4. **Discharge home on banana**:
   a. If discharge home on banana is desired, send detailed discharge recommendations home along with syringes to measure banana.
   b. Instructions should indicate storage for 48 hours in the refrigerator once opened and avoiding cross contamination.
   c. Family should demonstrate the ability to prepare thickened feeds using a syringe prior to discharge.
   d. The discharge summary should include clear written instructions including recipe, preparation, contraindications, and recommended follow-up.

**Anecdotal Notes:**

1. Chilled banana puree will be slightly thicker than room temperature banana puree and may have a slight enhanced thickening effect allowing slightly less banana to be utilized^4^
2. Banana may not thicken elemental formulas unless **true** IDDSI Levels 1 (“slightly thick”) or Level 2 (“Mildly thick”) are achieved. ^6^
3. The best thickening effect is seen with intact protein, hypoallergenic formula, and human milk.
4. Some elemental formulas may have a slight change in color to gray once banana is added, which has been clinically effective yet is visually not appealing and should be noted in discussions with team and family prior to use.
5. Infants generally have no overt GI bowel changes and often have improved frequency and consistency with small amounts of banana puree as indicated in the example recipes.
6. Documentation in EMR: Include banana as a homogeneous mixture to account for volume (e.g. 60ml of breastmilk with 9ml of banana to be recorded as 69ml and incorporated into targeted feed volume). ^6^ Do not exceed total volume ordered with NG in place.

**Appendix 1^[9]^**

**Guideline References**

1. Cichero, J.A., Nicholson, T.M. & September, C. (2013). Thickened milk for the management of feeding and swallowing issues in infants: A call for interdisciplinary professional guidelines. *Journal of Human Lactation*. 29 (2) 132-135. <https://doi.org/10.1177/0890334413480561>

2. McSweeney ME, Kerr J, Amirault J, Mitchell PD, Larson K, Rosen R. (2015). Oral feeding reduces hospitalizations compared with gastrostomy feeding in infants and children who aspirate. *Journal of Pediatrics.* 170:79–84. <https://doi.org/10.1016/j.jpeds.2015.11.028>

3.Duncan, D.R., Larson, K., Rosen, R.L. (2019). Clinical aspects of thickeners for pediatric gastroesophageal reflux and oropharyngeal dysphagia. *Gastroenterology Reports.* 21:30.

<https://doi.org/10.1007/s11894-019-0697-2>

4.Brooks, L., DiStefano, C. C., Clayton, H., & Gethers, C. T. (2024). Thickening human milk: the effect of time, temperature, and thickener for infants with dysphagia. *European journal of pediatrics,* 183(4), 1839–1848. <https://doi.org/10.1007/s00431-024-05434-5>

5.Brooks, L., Liao, J., Ford, J., Harmon, S., & Breedveld, V. (2022). Thickened Liquids Using Pureed Foods for Children with Dysphagia: IDDSI and rheology measurements. *Dysphagia,* 37(3), 578–590. <https://doi.org/10.1007/s00455-021-10308-1>

6.Brinker, K., Winn, L., Woodbury, A. E., Finch, A., Taggart, M., Thomas, D., Ermarth, A., & Chan, B. (2025). The nutrition profile and utility of banana puree as a liquid thickener for medically complex infants with dysphagia. *Nutrition in clinical practice: official publication of the American Society for Parenteral and Enteral Nutrition*, 40(1), 227–238. <https://doi.org/10.1002/ncp.11240>

7.Wolter, N.E., Hernandez, K., Irace, A.L., Davidson, K., Perez, J.A., Larson, K., Rahbar, R. (2018). A systematic process for weaning children with aspiration from thickened liquids. *JAMA Otolaryngology-Head&Neck Surgery*. 144(1):51-56. <https://doi.org/10.1001/jamaoto.2017.1917>

8.Balest, A.L., White, K.E., Shaffer, A.D., Mahoney, A.S., Georg, M., Theiss, R., & Dohar, J. (2020). Consideration of cough reflex development when ordering modified barium swallow studies in infants. *Dysphagia*. 35:533-541. <https://doi.org/10.1007/s00455-019-10062-5>.

Authored by: Sheri Ricciardi, OTR/L, CNT, SBCS

Owner: Sheri Ricciardi, Clinical Coordinator of Neonatal Therapy

Reviewed by: Banana QI Research Group, Pediatric Speech Language Pathology, Neonatal Occupational Therapy

Revised: March 20, 2025

1. Cichero, J.A., Nicholson, T.M. & September, C. (2013). Thickened milk for the management of feeding and swallowing issues in infants: A call for interdisciplinary professional guidelines. *Journal of Human Lactation*. 29 (2) 132-135. <https://doi.org/10.1177/0890334413480561>
2. McSweeney ME, Kerr J, Amirault J, Mitchell PD, Larson K, Rosen R. (2015). Oral feeding reduces hospitalizations compared with gastrostomy feeding in infants and children who aspirate. *Journal of Pediatrics.* 170:79–84. <https://doi.org/10.1016/j.jpeds.2015.11.028>
3. Duncan, D.R., Larson, K., Rosen, R.L. (2019). Clinical aspects of thickeners for pediatric gastroesophageal reflux and oropharyngeal dysphagia. *Gastroenterology Reports.* 21:30.

   <https://doi.org/10.1007/s11894-019-0697-2>
4. Brooks, L., DiStefano, C. C., Clayton, H., & Gethers, C. T. (2024). Thickening human milk: the effect of time, temperature, and thickener for infants with dysphagia. *European journal of pediatrics,* 183(4), 1839–1848. <https://doi.org/10.1007/s00431-024-05434-5>
5. Brooks, L., Liao, J., Ford, J., Harmon, S., & Breedveld, V. (2022). Thickened Liquids Using Pureed Foods for Children with Dysphagia: IDDSI and rheology measurements. *Dysphagia,* 37(3), 578–590. <https://doi.org/10.1007/s00455-021-10308-1>
6. Brinker, K., Winn, L., Woodbury, A. E., Finch, A., Taggart, M., Thomas, D., Ermarth, A., & Chan, B. (2025). The nutrition profile and utility of banana puree as a liquid thickener for medically complex infants with dysphagia. *Nutrition in clinical practice: official publication of the American Society for Parenteral and Enteral Nutrition*, 40(1), 227–238. <https://doi.org/10.1002/ncp.11240>
7. Balest, A.L., White, K.E., Shaffer, A.D., Mahoney, A.S., Georg, M., Theiss, R., & Dohar, J. (2020). Consideration of cough reflex development when ordering modified barium swallow studies in infants. *Dysphagia*. 35:533-541. <https://doi.org/10.1007/s00455-019-10062-5>.
8. Wolter, N.E., Hernandez, K., Irace, A.L., Davidson, K., Perez, J.A., Larson, K., Rahbar, R. (2018). A systematic process for weaning children with aspiration from thickened liquids. *JAMA Otolaryngology-Head&Neck Surgery*. 144(1):51-56. <https://doi.org/10.1001/jamaoto.2017.1917>
9. ^9^Brinker, K., Winn, L., Woodbury, A. E., Finch, A., Taggart, M., Thomas, D., Ermarth, A., & Chan, B. (2025). The nutrition profile and utility of banana puree as a liquid thickener for medically complex infants with dysphagia. *Nutrition in clinical practice: official publication of the American Society for Parenteral and Enteral Nutrition*, 40(1), 227–238. <https://doi.org/10.1002/ncp.11240>
